# Thymosin β_10_ Expression Driven by the Human TERT Promoter Induces Ovarian Cancer-Specific Apoptosis through ROS Production

**DOI:** 10.1371/journal.pone.0035399

**Published:** 2012-05-18

**Authors:** Young-Chae Kim, Byoung-Gie Kim, Je-Ho Lee

**Affiliations:** Department of Obstetrics and Gynecology, Samsung Medical Center, Molecular Therapy Research Center, Sungkyunkwan University School of Medicine, Gangnam-gu, Seoul, Korea; North Carolina State University, United States of America

## Abstract

Thymosin β_10_ (Tβ_10_) regulates actin dynamics as a cytoplasm G-actin sequestering protein. Previously, we have shown that Tβ_10_ diminishes tumor growth, angiogenesis, and proliferation by disrupting actin and by inhibiting Ras. However, little is known about its mechanism of action and biological function. In the present study, we establish a new gene therapy model using a genetically modified adenovirus, referred to as Ad.TERT.Tβ_10_, that can overexpress the Tβ_10_ gene in cancer cells. This was accomplished by replacing the native Tβ_10_ gene promoter with the human TERT promoter in Ad.TERT.Tβ_10_. We investigated the cancer suppression activity of Tβ_10_ and found that Ad.TERT.Tβ_10_ strikingly induced cancer-specific expression of Tβ_10_ as well as apoptosis in a co-culture model of human primary ovarian cancer cells and normal fibroblasts. Additionally, Ad.TERT.Tβ_10_ decreased mitochondrial membrane potential and increased reactive oxygen species (ROS) production. These effects were amplified by co-treatment with anticancer drugs, such as paclitaxel and cisplatin. These findings indicate that the rise in ROS production due to actin disruption by Tβ_10_ overexpression increases apoptosis of human ovarian cancer cells. Indeed, the cancer-specific overexpression of Tβ_10_ by Ad.TERT.Tβ_10_ could be a valuable anti-cancer therapeutic for the treatment of ovarian cancer without toxicity to normal cells.

## Introduction

Thymosins are a family of small proteins that were originally isolated from calf thymus, and are divided into three classes (α, β and γ) based on their isoelectric point [1]. The β-thymosins, which have an average molecular mass of roughly 5 kDa, are highly conserved acidic proteins that are found in almost every eukaryotic cell. The β-thymosins inhibit barbed end actin polymerization by sequestering actin monomers [2], [3]. As one of the most abundant β-thymosins in mammalian species, thymosin β_10_ (Tβ_10_) affects metastasis and proliferation in many cancer cells [4]–[6]. The anti-cancer effects of Tβ_10_ appear to be closely related to its function as a regulator of actin dynamics in tumor cells [7], [8]. Actin dynamics can be perturbed by the addition of actin-stabilizing drugs or the introduction of mutations, causing changes in cellular architecture and internal cellular movement. Furthermore, recent reports have indicated that changes in actin dynamics can result in the release of reactive oxygen species (ROS) from mitochondria and subsequent cell death, emphasizing the importance of maintaining the dynamic regulation of the actin cytoskeleton [9]–[14].

Recently, telomerase has been recognized as a wide-range tumor marker and is now considered one of the most important therapeutic targets for cancer treatment. Human telomerase reverse transcriptase (hTERT), the catalytic subunit of telomerase, is detected in approximately 90% of cancer cells from tumor tissue, but is not detectable in normal tissues [15]–[17]. Previous studies have demonstrated that the hTERT promoter can regulate the ectopic expression of genes of interest in telomerase-positive cancer cells, indicating that the hTERT promoter is a promising candidate for generating cancer-specific adenoviruses [18]–[22].

Here, we describe a recombinant adenovirus, Ad.TERT.Tβ_10_, that was constructed by inserting the Tβ_10_ gene under the control of the hTERT gene promoter into the adenovirus p-shuttle plasmid to induce tumor-specific Tβ_10_ gene expression. We also established a co-culture model of primary human ovarian cancer cells and normal fibroblasts and subsequently treated this co-culture with Ad.TERT.Tβ_10_ to elucidate the cancer-specific effects of Ad.TERT.Tβ_10_. In addition, we investigated the mechanism of Tβ_10_-induced apoptosis in 2774 human ovarian cancer cells that were treated with Ad.TERT.Tβ_10_. These experiments revealed evidence that Ad.TERT.Tβ_10_ induces cancer-specific expression of Tβ_10_, thereby resulting in cancer-specific apoptosis through ROS production. Together these findings indicate that the cancer-specific overexpression of Tβ_10_ by Ad.TERT.Tβ_10_ could indeed be a valuable anti-cancer therapeutic for the treatment of ovarian cancer without toxicity to normal cells and possibly other malignancies.

## Results

### Thymosin β_10_ is Expressed at Low Levels in Ovarian Cancer

**Figure 1:**
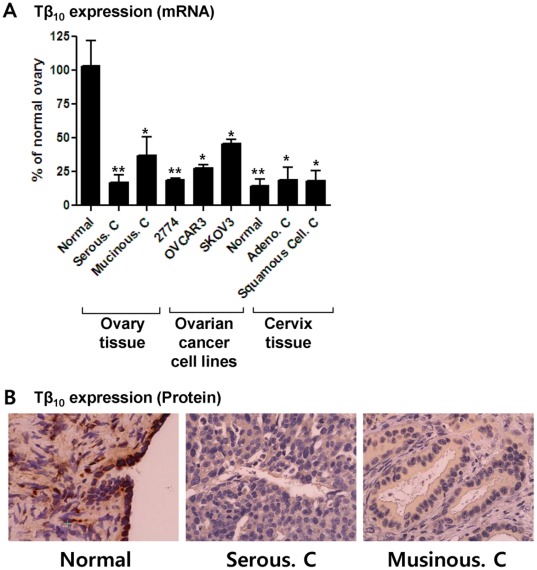
Comparison of endogenous mRNA and protein expression in the human ovary, cervix, and various cancer cell types. (A) mRNA expression levels of Tβ_10_ were determined by real-time PCR using total RNA isolated from human normal and cancer tissues including ovarian cancer cell lines, such as serous carcinoma (Serous. C) and mucinous carcinoma (Mucinous. C), immortalized human ovarian cancer cell lines (2774, OVCAR3, and SKOV3), and adenocarcinoma (Adeno. C) and squamous cell carcinomas (Squamous Cell. C) of the cervix. Data is presented as mean ± SE for n = 3 separate samples per cell type grouping and is expressed in terms of percentage of normal ovary. Significant differences between groups are denoted by an asterisk (*), P<0.05; a double asterisk (**), P<0.01 (B) The protein expression level of Tβ_10_ was determined by immunohistochemistry in normal ovary tissue and compared with that in serous and mucinous types of ovarian cancer, respectively.

In our previous study, we reported that Tβ_10_ mRNA levels were elevated in normal ovaries, as compared to other tissues, such as spleen, thymus, prostate, testis, small intestine, colon, and peripheral blood leukocytes, but the mRNA levels of Tβ_10_ were decreased in ovarian cancers [23]. We, therefore, confirmed the mRNA and protein expression levels of thymosin β_10_ (Tβ_10_) in serous type ovarian cancer and mucinous type ovarian cancer, as well as in cervical cancer and immortalized ovarian cancer cell lines, such as 2774, OVCAR3, and SKOV3. Our findings that mRNA (Figure 1A) and protein (Figure 1B) levels of Tβ_10_ were high in normal ovarian tissue, but decreased in all of Serous Carcinoma, Mucinous Carcinoma, and the immortalized ovarian cancer cell lines, were consistent with our previous study. However, we were unable to detect changes in normal cervical tissue, adenocarcinomas, and squamous cell carcinomas. Notably, high expression levels of Tβ_10_ are characteristic of normal ovaries, but these levels decrease in ovarian cancer, suggesting that Tβ_10_ plays a role in ovarian cancer development.

### Cancer-specific Expression of Thymosin β_10_ by Ad.TERT.Tβ_10_

**Figure 2:**
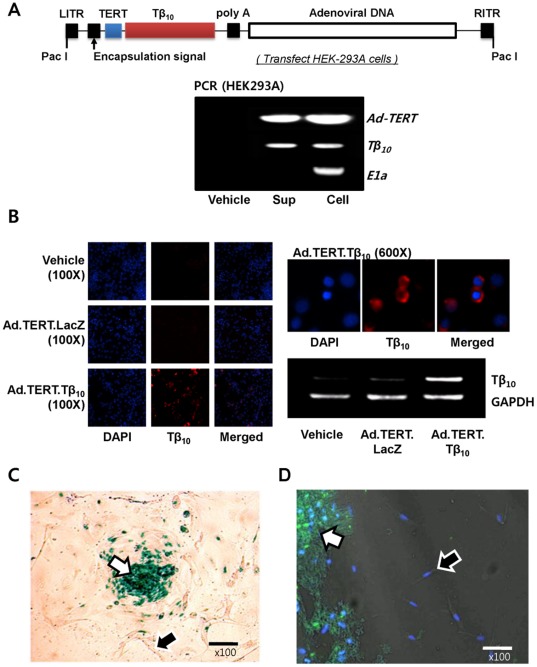
Construction of a recombinant adenovirus vector based on the AdEasy™ system and confirmation of cancer-specific gene introduction by the recombinant adenovirus (Ad.TERT.Tβ_10_). (A) Schematic diagram of the recombinant adenovirus, Ad.TERT.Tβ_10_, generated by recombination of pSuttle.TERT.Tβ_10_ and the backbone plasmid pAdEasy-1 in BJ5183. The modified pShuttle plasmid, referred to as pSuttle.TERT.Tβ_10_, was cloned by orderly insertion of the human TERT promoter and Tβ_10_ gene to generate a recombinant adenovirus. The integrity of Ad.TERT.Tβ_10_ was determined by polymerase chain reaction (PCR) using primers Ad-TERT, Tβ_10_, and E1a. (B) Subconfluent 2774 ovarian cancer cells were infected with 10 MOI of Ad.TERT.LacZ or Ad.TERT.Tβ_10_. After 24 hr, Tβ_10_ protein and mRNA levels were determined by immunocytochemistry and RT-PCR, respectively. (C) Subconfluent primary human ovarian cancer cells co-cultured with fibroblast cells were exposed to 10 MOI of Ad.TERT.LacZ for 7 days. β-galactosidase expression level was determined by β-gal staining (blue color). Arrow at center of figure indicates a primary ovarian cancer cell mass. (D) Subconfluent primary human ovarian cancer cells co-cultured with fibroblast cells were also exposed to 10 MOI of Ad.TERT.Tβ_10_ for 24 hr. Tβ_10_ expression level was determined by immunocytochemistry (green color). Arrows at the bottom of figure indicate primary ovarian cancer cells.

To elucidate the role of Tβ_10_ in human ovarian cancer, we established a new physiological model by co-culturing primary ovarian cancer cells with normal fibroblasts and using a cancer-specific gene delivery system comprising a genetically modified adenovirus. We constructed recombinant adenovirus, Ad.TERT.Tβ_10_, by replacing the Tβ_10_ gene promoter with the hTERT promoter, and we confirmed the integrity of Ad.TERT.Tβ_10_ by polymerase chain reaction (PCR) using Ad-TERT, Tβ_10_, and E1a primers (Figure 2A). To confirm the level of Tβ_10_ gene expression, we treated 2774 cells with Ad.TERT.Tβ_10_ at a multiplicity of infection (MOI) of 10 and then monitored changes in the Tβ_10_ expression using immunocytochemistry (ICC) and RT-PCR. Our results revealed that Ad.TERT.Tβ_10_ elevated Tβ_10_ expression in the cytosol (Figure 2B).

We also evaluated cancer-selective induction of gene expression by recombinant adenoviruses, Ad.TERT.Tβ_10_ and Ad.TERT.LacZ, on our co-culture model of primary ovarian cancer cells and normal fibroblasts. On day 7 after treatment with 10 MOI of Ad.TERT.LacZ, we observed that ovarian cancer cells had grown well on the layer formed by normal fibroblast cells like being observed in untreated group. Furthermore, X-gal positive cells, indicating β-galactosidase expression by Ad.TERT.LacZ, were detected only in primary ovarian cancer cells (Figure 2C). On day 1 after treatment with Ad.TERT.Tβ_10_, green fluorescence, indicating Tβ_10_ expression, was detected in primary ovarian cancer cells but not in normal fibroblast cells (Figure 2D), suggesting that the TERT promoter confers ovarian cancer-specific gene expression.

### Effects of Thymosin β_10_ on Ovarian Cancer Cell Migration

**Figure 3:**
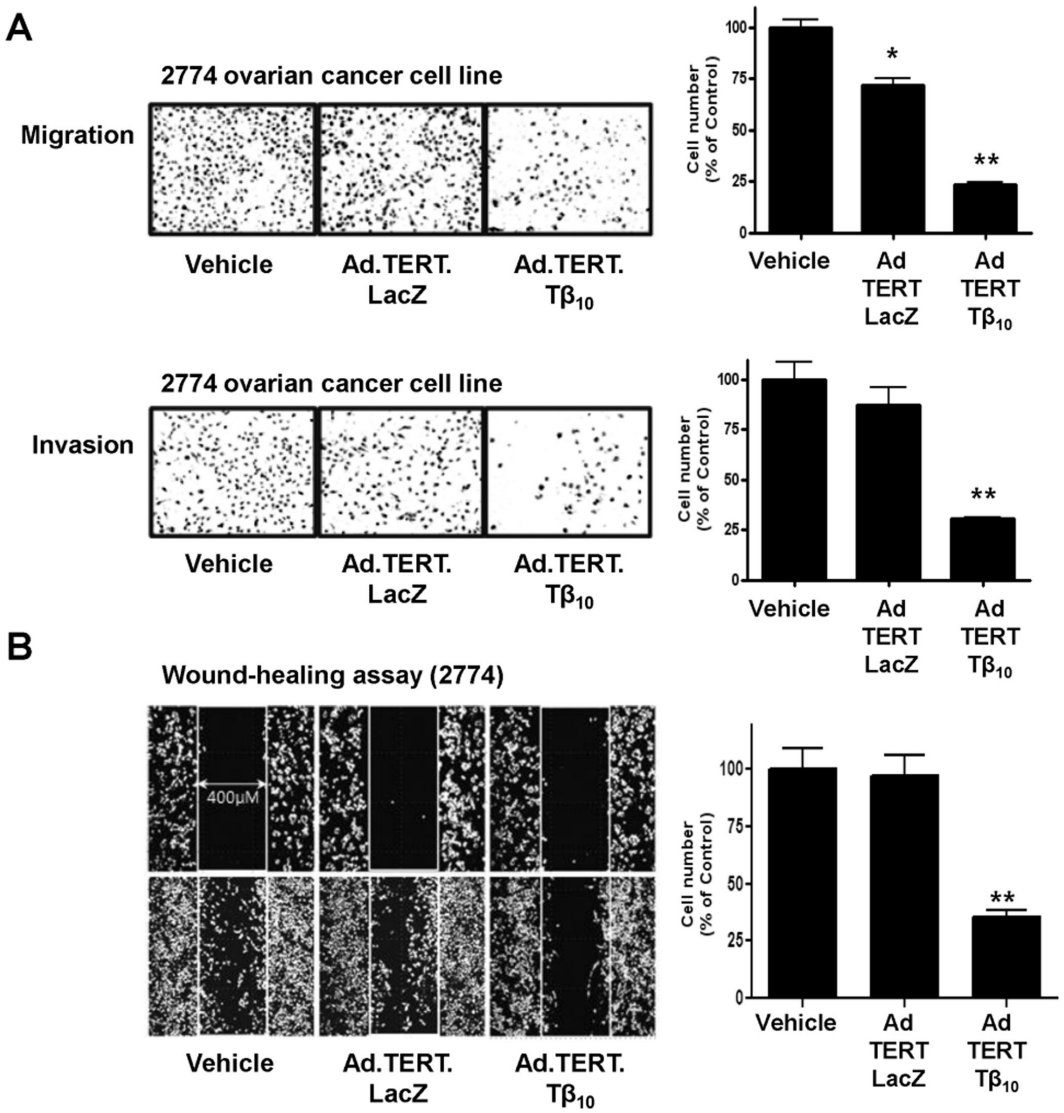
The decrease in 2774 ovarian cancer cell migration and invasion as a result of Tβ_10_ overexpression. (A) Subconfluent 2774 human ovarian cancer cells were infected with 10 MOI of Ad.TERT.LacZ or Ad.TERT.Tβ_10_ for 12 hr. After re-suspension, cells were further incubated for 12 hr in the upper chamber of a Transwell® plate coated with a collagen monolayer for the migration assay or with double layer of collagen and matrigel for the invasion assay. The results are expressed as a percentage of the vehicle control and are mean ± SE (bars) of three experiments. Significant differences from vehicle control group are denoted by an asterisk (*), P<0.05; a double asterisk (**), P<0.01 (B) The virus-infected cells were also further incubated in a 35 mm µ–Dish, low, with a culture-Insert (Ibidi) for 12 hr, and followed by observation of the direction and speed of cell migration during wound-healing. The wound-healing results are expressed as a percentage of the vehicle control and are mean ± SE (bars) of three experiments. Significant differences from vehicle control group are denoted by a double asterisk (**), P<0.01.

Previous reports demonstrated that the anti-cancer effects of Tβ_10_ are closely related to its role in regulating actin dynamics in tumor cells [7], [8]. We, therefore, examined the role of Tβ_10_ in cancer cell migration and invasion. We found that both cancer cell migration and invasion were significantly decreased in the 2774 cells infected by Ad.TERT.Tβ_10_, in comparison with 2774 cells infected with Ad.TERT.LacZ and uninfected 2774 cells (Figure 3A). In relation to cell migration, cell movement was also estimated by wound-healing assay. 2774 cell motility on a plastic surface was significantly reduced by Ad.TERT.Tβ_10_ treatment (Figure 3B).

### Induction of Cancer-specific Apoptosis by Ad.TERT.Thymosin β_10_

**Figure 4:**
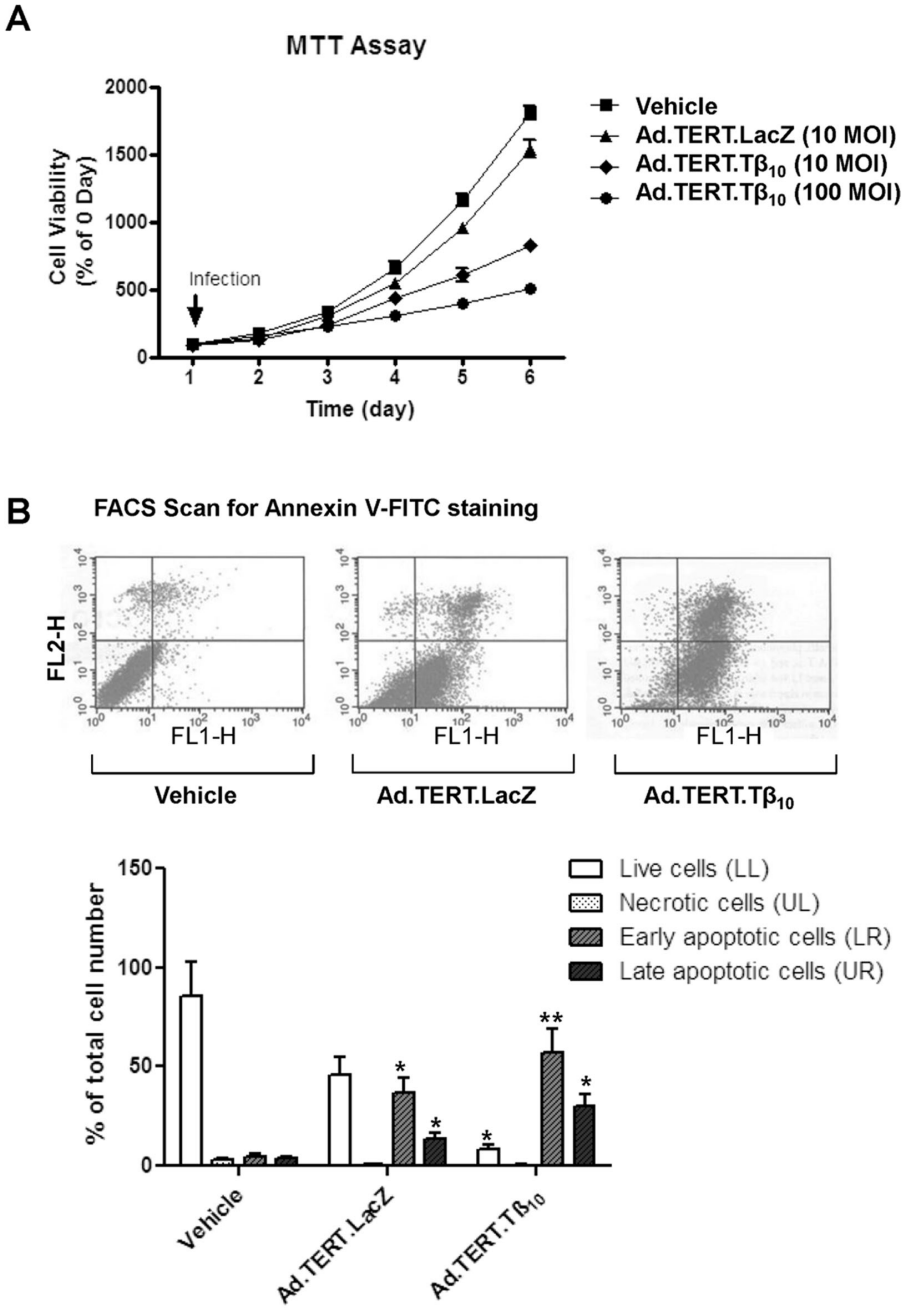
Induction of apoptosis by overexpression of Tβ_10_ in 2774 ovarian cancer cells. (A) Subconfluent 2774 ovarian cancer cells were maintained for 5 days in complete culture medium containing Ad.TERT.LacZ or Ad.TERT.Tβ_10_ at an MOI of 10 or 100, and the MTT-bioassay was performed daily. (B) On the second day after viral infection, a FACS Annexin V/propidium iodide (PI) assay was performed according to the manufacturer’s (Clontech) protocol. Representative scatter plots are showing the distribution of annexin V- and PI-stained cells. In top panels, the X-axis (FL1-H) indicates annexin V–FITC fluorescence detected at 518 nm, and the Y-axis (FL2-H) indicates PI fluorescence detected at 620 nm. As the lower left (LL) quadrant indicates live cells, the upper left (UL) quadrants indicate necrotic cells, the upper right (UR) quadrats indicate late apoptotic cells, and lower right (LR) quadrant indicates early apoptotic cells (top panels), the results are expressed as a percentage of total cell numbers and are mean ± SE (bars) of three individual experiments. Significant differences from vehicle group are denoted by an asterisk (*), P<0.05; a double asterisk (**), P<0.01.

**Figure 5:**
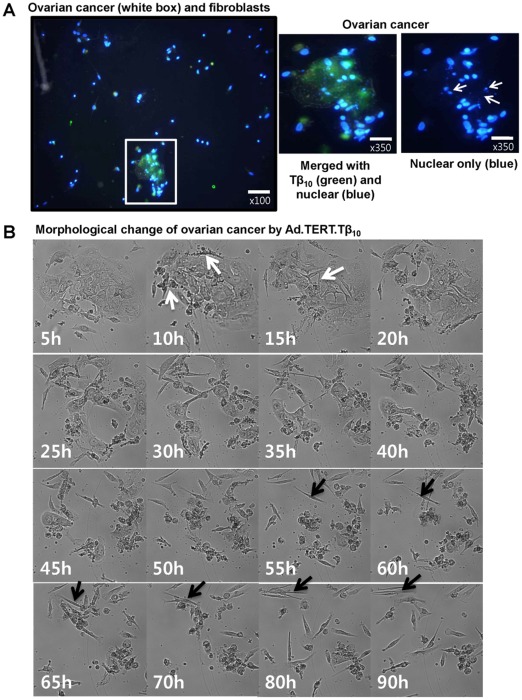
Ad.TERT.Tβ_10_-induced selective apoptotic morphological changes in human primary ovarian cancer cells, but not in normal fibroblasts. (A) Co-cultures of subconfluent primary human ovarian cancer cells and fibroblasts were exposed to 10 MOI of Ad.TERT.Tβ_10_ for 24 hr, and then immunocytochemistry was performed to observe the nuclear morphological changes induced by Tβ_10_ expression. Differential interference contrast (DIC) image was merged with 4,6-diamidino-2-phenylindole (DAPI) staining (blue) and Alexa 488 staining (green) to evaluate nuclear fragmentation by Tβ_10_ expression. Arrows indicate nuclear fragmentations. (B) Co-cultured subconfluent primary human ovarian cancer cells and fibroblasts were exposed to 10 MOI of Ad.TERT.Tβ_10_ for over 90 hr, and morphological changes were then observed using a real-time imaging system. White arrows indicate selective morphological features of apoptosis, such as cell plasma membrane blebbing, cytoplasmic vacuolization, and cell shrinkage (after 10 hr), followed by loss of cell-to-cell interactions (after 15 hr). And black arrows tracking one of fibroblasts indicate that fibroblasts are actively moving about even until 90 hr.

To examine the effects of overexpression of Tβ_10_ on cancer cell viability, subconfluent 2774 ovarian cancer cells were infected with Ad.TERT.LacZ or Ad.TERT.Tβ_10_ at an MOI of 10, and the MTT-bioassay was performed daily for 6 days. We found that Tβ_10_ significantly decreased ovarian cancer cell viability, when compared with media controls with or without Ad.TERT.LacZ (Figure 4A). Next, to examine whether the decrease in cell viability was due to apoptosis, we performed FACS analysis for Annexin V-FITC and found that overexpression of Tβ_10_ induced apoptosis, but not necrosis, in the 2774 ovarian cancer cell line (Figure 4B). Moreover, on day 1 after treatment of Ad.TERT.Tβ_10_ in co-culture model of primary human ovarian cancer with normal fibroblasts, we clearly observed nuclear fragmentation (blue fluorescence) only in ovarian cancer cells that overexpressed Tβ_10_ (green fluorescence). Moreover, we were unable to detect nuclear fragmentation or Tβ_10_ expression above background levels the normal fibroblast. These results indicate that cancer-specific overexpression of Tβ_10_, which is facilitated by the TERT promoter, stimulates cancer-specific nuclear fragmentation as one of the morphological hallmarks of apoptosis (Figure 5A). In addition we observed striking morphological changes of ovarian cancer cells treated with Ad.TERT.Tβ_10_ for over 90 hr using a real-time imaging microscope. After 10 hours of treatment with Ad.TERT.Tβ_10_, we observed typical apoptotic morphological changes in the ovarian cancer cells such as plasma membrane blebbing, loss of cell-cell interactions, cytoplasmic vacuolization, and cell shrinkage due to the rapid dehydration. Apoptotic cells gradually disappeared by cell death, whereas normal fibroblast cells seemed to be unaffected by Ad.TERT.Tβ_10_ for over 90 hr (Figure 5B; Movie S1).

### The Relationship Between Thymosin β_10_ and Fas Expression

**Figure 6:**
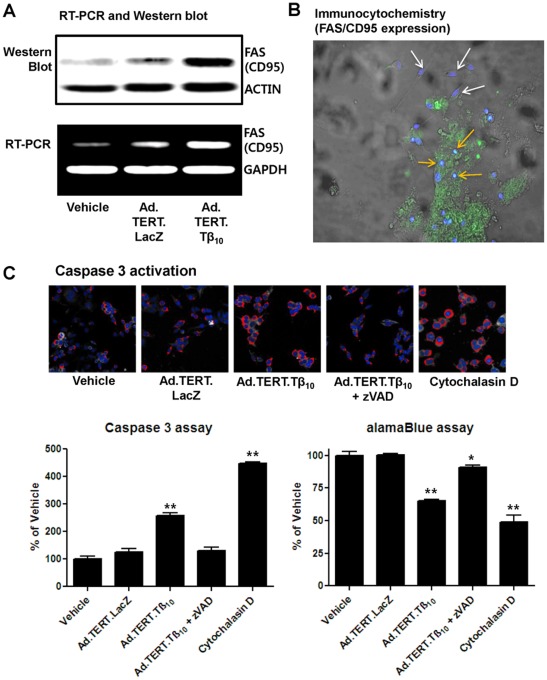
Ad.TERT.Tβ_10_-induced increase in cancer-specific FAS expression in human ovarian cancer cells, resulting in apoptosis. (A) Subconfluent 2774 human ovarian cancer cells were exposed to Ad.TERT.LacZ or Ad.TERT.Tβ_10_ at an MOI of 10 for 48 hr and followed by next experiments such as RT-PCR and Western blotting to detect FAS (CD95) mRNA and protein expression, respectively. (B) Subconfluent primary human ovarian cancer cells co-cultured with fibroblasts were also exposed to 10 MOI of Ad.TERT.Tβ_10_ for 24 hr and followed by immunocytochemistry. Specific FAS/CD95 antibody staining (Alexa 488, green) was merged with differential interference contrast (DIC) images and nuclear staining images (DAPI, blue) using imaging software. White and orange arrows indicate fibroblasts and ovarian cancer cells, respectively. (C) Cells were exposed to Ad.TERT.LacZ or Ad.TERT.Tβ_10_ at an MOI of 10 with or without caspase inhibitor (zVAD, 40 µM), and actin polymerization inhibitor (cytochalasin D, 1 µg/ml) for 48 hr. The 2774 ovarian cancer cells were then stained according to the commercial caspase 3 assay kit protocol (Thermo) and imaged using a Thermo Scientific Cellomics ArrayScan high-content screening (HCS) reader. In the bar graph, the values representing a percentage of the vehicle control is followed by the ratio between the area of caspase 3 activation and the area of the nucleus. And the results are mean ± SE (bars) of three different experiments. Significant differences from vehicle group are denoted by a double asterisk (**), P<0.01 (left bar graph). Cytotoxicity under the same conditions was determined in cultured ovarian cancer cells using the alamar Blue (AB) assay as an *in vitro* alternative model. The results are expressed as a percentage of the vehicle control and are mean ± SE (bars) of three experiments. Significant differences from vehicle control group are denoted by an asterisk (*), P<0.05; a double asterisk (**), P<0.01 (right bar graph)

To examine whether ovarian cancer-specific apoptosis is due to apoptosis-promoting gene expression and subsequent signal transduction, we assessed Fas expression and caspase 3 activation using primary ovarian cancer cells co-cultured with normal fibroblasts as well as the 2774 ovarian cancer cell line. We found that Ad.TERT.Tβ_10_ induced Fas mRNA and protein expression in 2774 ovarian cancer cells (Figure 6A). In the primary co-culture model, Fas expression was detected in primary ovarian cancer cells but not in normal fibroblasts. These findings are consistent with the cancer-specific expression of Tβ_10_ after infection with Ad.TERT.Tβ_10_ (Figure 6B). Importantly, Tβ_10_ increased caspase 3 activity as did Cytochalasin D (Cyt D), a potent inhibitor of actin polymerization. The increased caspase 3 activity was also inhibited by the addition of caspase inhibitor Z-VAD-FMK. Furthermore, these data are consistent with the cell viability results obtained from the alarmar Blue assay (Figure 6C).

### The Effects of Thymosin β_10_ on Mitochondrial Dysfunction and ROS Production

**Figure 7:**
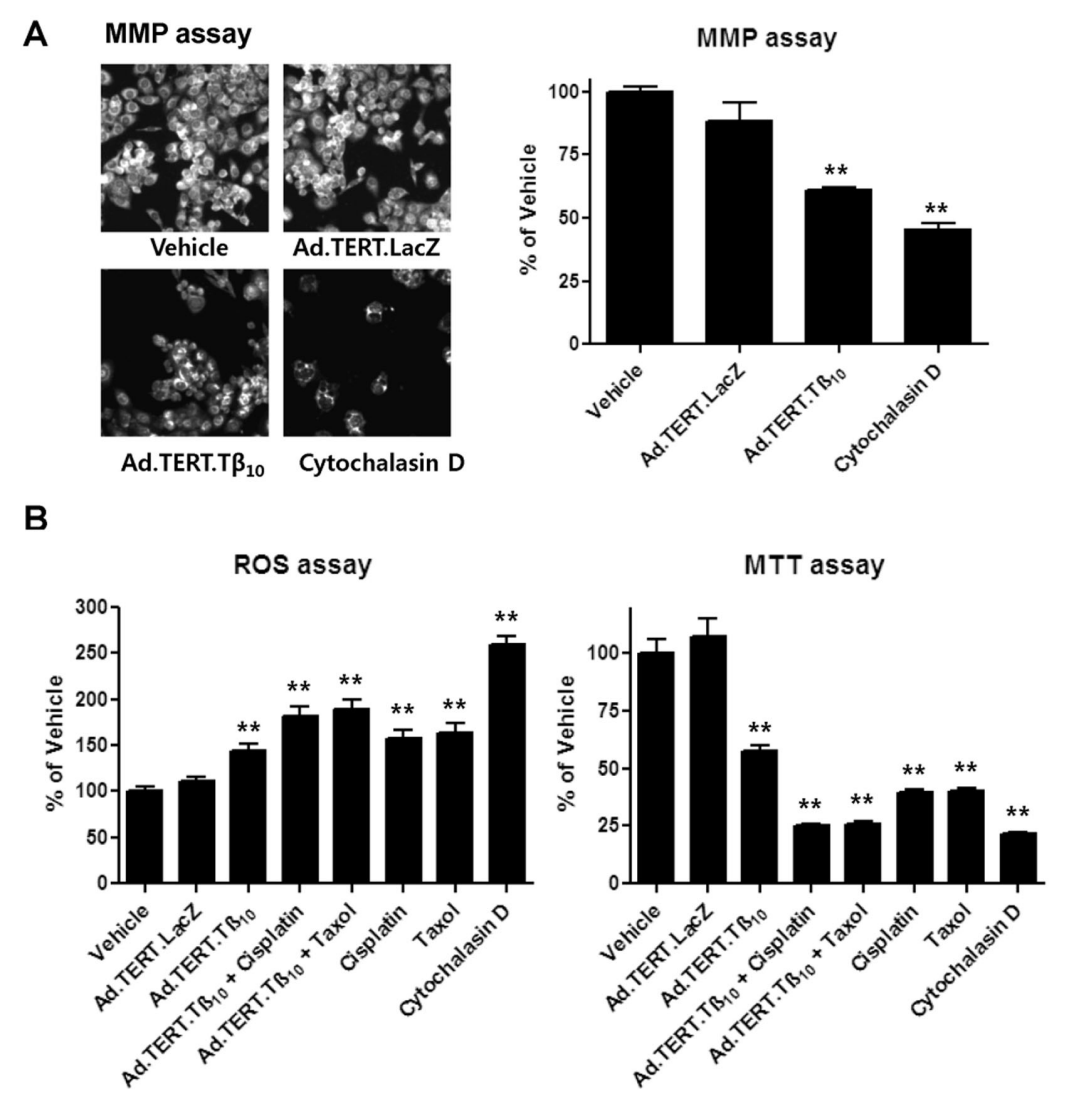
Ad.TERT.Tβ_10_-induced decrease in mitochondria membrane potential (MMP) and increase in ROS production in 2774 human ovarian cancer cells. (A) Cells were exposed to Ad.TERT.LacZ (10 MOI), Ad.TERT.Tβ_10_ (10 MOI), and actin polymerization inhibitor (cytochalasin D, 1 µg/ml) for 48 hr, and then a mitochondrial membrane potential (MMP) assay was performed. The MMP assay was performed using the MMP assay kit protocol (Thermo), and the results were imaged using a Thermo Scientific Cellomics ArrayScan high-content screening (HCS) reader. (B) Cells were exposed to Ad.TERT.LacZ (10 MOI), Ad.TERT.Tβ_10_ (10 MOI) with or without anti-cancer agents, such as paclitaxel (taxol, 5 µg/ml) and cisplatin (platinum, 10 µg/ml), for 48 hr. The results were compared to those from other treatments, for example, each anti-cancer agent or actin polymerization inhibitor (cytochalasin D, 1 µg/ml). The levels of intracellular reactive oxygen species (ROS) were detected by a fluorescence ELISA system with the oxidation-sensitive fluorescent probe 2, 7-dichlorofluorescein diacetate (DCF-DA). In addition to the ROS assay, an MTT-bio assay was also performed under the same conditions. The all results are expressed as a percentage of the vehicle control and are mean ± SE (bars) of three experiments. Significant differences from vehicle control group are denoted by an asterisk (*), P<0.05; a double asterisk (**), P<0.01.

Previous reports have indicated that disruption of the actin cytoskeleton induces the extracellular generation of ROS, in particular O_2_^-^, which amplifies Fas-dependent apoptosis [13], [24]. In addition, decreased actin dynamics cause depolarization of the mitochondrial membrane and an increase in ROS production, resulting in cell death [14]. Taken together, we hypothesized that inhibition of actin polymerization by Tβ_10_ opens mitochondrial membrane pores or channels for a prolonged time, resulting in reduction of the mitochondrial membrane potential (MMP) and an increase in the release of ROS into the cytoplasm. Therefore, we measured Tβ_10_-induced changes in MMP ROS levels and found that initially high levels of MMP were markedly decreased by Tβ_10_ overexpression (Figure 7A). Moreover, ROS production was increased by Tβ_10_ overexpression, which is consistent with the result of the MTT bioassay.

It has been reported that cellular ROS levels increase under conditions of stress by treatment with anticancer agents such as taxol (paclitaxel) and platinum (cisplatin). This ROS increase leads to the induction of proapoptotic molecules, such as p53 and mitogen-activated protein kinases (MAPKs), leading to cellular apoptosis [25]–[30]. We used Ad.TERT.Tβ_10_ as a co-treatment with two classes of the anticancer agents, and found that the co-treatment resulted in synergistic effects, increasing ROS generation and decreasing cell viability (figure 7B). These findings suggest that ovarian cancer apoptosis induced by Tβ_10_ overexpression is due to ROS production in response to decreased MMP.

## Discussion

Of the ß-thymosin family members, thymosin ß_4_ (Tβ_4_), thymosin ß_10_ (Tβ_10_), and thymosin ß_15_ (Tβ_15_) have been studied in carcinogenesis. They have been implicated in actin sequestration. However, the function of β-thymosins is largely unknown, and their role in neoplastic disease progression remains controversial.

The elevated expression of Tβ_4_ and Tβ_10_ has been regarded as a characteristic of human carcinogenesis. Overexpression of Tβ_4_ occurs in a wide variety of human carcinomas. Tβ_4_ affects angiogenesis and cancer cell migration in many cancer cell types [31]–[34]. Likewise, overexpression of Tβ_10_ has been reported in numerous human carcinomas, including melanomas [1], carcinomas of the kidney, pancreas [35], and stomach [36], as well as medullary thyroid carcinomas [37].

Paradoxically, Tβ_4_ and Tβ_10_ may be involved in cancer degeneration, as reports have indicated that Tβ_4_ has tumor suppressive effects in myeloma [38], and Tβ_10_ is deregulated in renal cell carcinoma [39] and ovarian cancer [40]. In addition, Tβ_10_ inhibited cell migration and capillary-like tube formation of human coronary artery endothelial cells [6]. Our findings also implicate Tβ_10_ in cancer degeneration, as the high level of Tβ_10_ transcripts in normal ovary was remarkably reduced in ovarian carcinomas. Moreover, ectopic expression of Tβ_10_ in ovarian carcinoma cell lines, reduced cellular proliferation and movement, and apoptosis was accelerated. Hence, Tβ_10_ overexpression in normal ovarian cells appears to act as a negative regulator of carcinogenesis, though the exact mechanism is unclear. However, as Tβ_4_ is induced in ovarian cancer [34] and plays a role in actin sequestration like Tβ_10_, ectopic expression of Tβ_10_ in our experimental system may lead to increased activity of ß-thymosins and subsequent perturbation of actin dynamics. This concept is supported by reports that Tβ_4_ and Tβ_10_ act on vessel development in a complementary way in vivo [6] and that the imbalance of G-actin and F-actin causes cell rounding and death [41]. Thus, we considered a possible strategy for cancer research and gene therapy with Tβ_10_. We, therefore, manufactured a recombinant adenovirus, Ad.TERT.Tβ_10_, to use for human gene therapy and to elucidate the mechanism of Tβ_10_ in ovarian cancer prevention.

Recombinant adenovirus systems are versatile for gene expression studies and therapeutic applications, and many researchers have used recombinant adenoviruses with great success over the years. However, their low specificity is a disadvantage limiting the usefulness of these vectors as *in vivo* therapeutic agents. Vectors that combine high infectivity with cancer cell-specific expression are, therefore, needed. Several studies have shown that human telomerase reverse transcriptase (hTERT) is highly expressed in most tumor tissues but not in normal tissues. For example, hTERT promoter activity is higher in human and murine cancer cells derived from lung, colon, liver, breast, ovary, and brain [42]–[47]. Conversely, they have shown that the hTERT promoter is inactive in normal human fibroblasts [42], and normal human epithelial cells from trachea [42], mammary [47], and ovary [45], [48]. Therefore, we examined the role of Tβ_10_ in ovarian cancer using a recombinant adenovirus (Ad.TERT.Tβ_10_). Ad.TERT.Tβ_10_ was constructed by insertion of the Tβ_10_ gene under the control of the hTERT promoter into the adenovirus p-shuttle plasmid to obtain cancer-specific expression, and it was compared with Ad.TERT.LacZ as a control. In a co-culture model of primary human ovarian cancer cells and normal fibroblasts, β-galactosidase driven by the hTERT promoter was expressed in ovarian cancer cells only but not in normal fibroblasts. Cytotoxic and apoptotic morphological changes were absent from the normal fibroblasts treated with 10 MOI of Ad.TERT.LacZ over a 7-day period, in contrast to the ovarian cancer-specific apoptosis induced by treatment of cells with 10 MOI of Ad.TERT.Tβ_10_ over a 10-hour period. Moreover, we found that the cancer-specific expression of Tβ_10_ from Ad.TERT.Tβ_10_ was accompanied by the elevated expression of Fas, resulting in cancer-specific apoptosis. Therefore, we considered that the anticancer effect of Ad.TERT.Tβ_10_ appeared to be associated with the overexpression of Tβ_10_ rather than non-specific viral toxicity.

Previous studies have reported that a high level of ROS has a positive effect on cancer development and proliferation [49]–[56]. However, we found that the actin sequestration by overexpression of Tβ_10_ was due to the mitochondrial dysfunction and ROS elevation, by which apoptosis signaling was activated. The idea that cancer cells are vulnerable to attack by ROS is supported by the therapeutic use of widely accepted anticancer drugs such as taxol and cisplatin. Both of these drugs increase ROS generation in 2774, and cotreatment of anticancer agents with Tβ_10_ result in an additive effect of ROS elevation and subsequent induction of apoptosis. Therefore, the fate of the cancer cell seems to be determined by the ROS balance. Mild elevation of ROS may help cancer development, but excessive ROS elevation in cancer cells under some stresses such as Tβ_10_ overexpression and treatment of anticancer drugs appears to stimulate apoptotic signals.

In addition to induction of apoptosis, Tβ_10_ overexpression potently reduced motility in the 2774 ovarian cancer cells, and this effect was independent of apoptosis induction. In the 2774 ovarian cancer cells, apoptosis was induced much later than in primary ovarian cancer cells. Ad.TERT.Tβ_10_-induced apoptosis in primary ovarian cancer cells and immortalized cancer cell lines such as 2774, OVCAR3, and SKOV3, occurred 10- and 48-hours later, respectively. As the assays of invasion and migration were performed prior to observation of apoptosis, we suggest that Tβ_10_ overexpression in ovarian cancer may have beneficial cell-killing effects of primary tumor cells as well as metastatic cells.

Our findings indicate that the anti-cancer activity of Tβ_10_ may be due to ROS generation. Furthermore, Tβ_10_ and anti-cancer agents, such as taxol and cisplatin, had a synergistic effect on cancer cell death. We also found that cancer-specific overexpression of Tβ_10_ driven by the hTERT promoter resulted in cancer-selective apoptosis through amplification of FAS signaling. This study provides insight into the mechanism underlying the anti-cancer effects of Tβ_10_ in ovary, which may prove useful for the development of effective ovarian cancer therapies without side effects.

## Materials and Methods

### Ethics Statement

The works using human sample were received the written consent of a patient and approved by the Korean Association of Institutional Review Board (KAIRB).

### Cell Lines and Primary Cell Culture

The human ovarian cancer cell line 2774 was obtained from the American Type Culture Collection (ATCC), and primary human ovarian cancer and fibroblast cells were isolated from patients with grade III endometrioid adenocarcinoma (cancer) and co-cultured on glass surfaces. Cells were grown in Dulbecco’s modified Eagle’s medium (DMEM; Invitrogen, Carlsbad, CA, USA) supplemented with 10% fetal bovine serum (FBS; Upstate Biotechnology, Lake Placid, NY, USA), and 100 U/ml penicillin/streptomycin (Invitrogen).

### Viral Vectors

The recombinant adenovirus, “Ad.TERT.Tβ_10_,” was constructed according to the standard procedure used in the AdEasy™ Adenoviral Vector System (Stratagene, La Jolla, CA, USA). The recombinant adenovirus Ad.TERT.Tβ_10_ plasmid was generated by recombination of the modified pShuttle plasmid (pSuttle.TERT.Tβ_10_) and backbone plasmid pAdEasy-1 in *E.coli* BJ5183. Subsequently, the recombinant adenoviral plasmid DNA was isolated using a plasmid DNA isolation kit (Axygen, Union City, CA, USA), linearized by a Pac I restriction enzyme digest, and then transfected into 293A cells. The manufactured recombinant adenovirus was purified using an adenovirus purification kit (Cell Biolabs, San Diego, CA, USA).

### Reverse Transcription Polymerase Chain Reaction (PCR), and Real-time PCR

Cells were washed twice with ice-cold PBS, and the total RNA was extracted from the samples with Trizol (Invitrogen). Reverse transcription (RT) was performed using 1 µg total RNA and the SuperScript™ II RT kit (Invitrogen). First-strand cDNA synthesis from total RNA was completed by heating for 60 min at 42°C and 15 min at 70°C. One microliter of RT reaction mixture was then amplified using the Taq polymerase kit (Invitrogen) to produce cDNA using an iCycler PCR detection system (Bio-Rad Laboratories, CA, USA). Furthermore, 10 µl of RT reaction mixture was used as a template for RT-PCR using the iQ™ SYBR® Green Supermix kit (Bio-Rad, Invitrogen) using an iCycler iQ real-time PCR detection system (Bio-Rad). The primers used for PCR were as follows: Ad-TERT upstream primer, 5′-AAATTTGGGCGTAACCGAGT-3′; Ad-TERT downstream primer, 5′-ATGAGGCCAACATCTGGTCA-3′; Tβ_10_ upstream primer, 5′-GTCGACATGGCAGACAAACCAGA-3′; Tβ_10_ downstream primer, 5′-GATGGACACGAGCCACAAAGATCT-3′; Ad-E1a upstream primer, 5′-CGGGATCCCCACCATGAGACATATTATCTGCCACG-3′; Ad-E1a downstream primer, 5′-CGGAATTCTTACTCGAGGTCAATCCCTTCCTGCACC-3′; FAS/CD95 upstream primer, 5′-CTCCAACCTTAAATCCTGAAACAG-3′; and FAS/CD95 downstream primer, 5′-CGAACAAAGCCTTTAACTTGACTT-3′. The PCR procedure comprised a denaturing step of 94°C for 3 min is followed by 30 cycles of denaturation at 94°C for 1 min, annealing at a primer pair-specific temperature (Ad.TERT, 53°C; Tβ_10_, 61°C; Ad-E1a, 44°C; FAS/CD95, 53°C) for 1 min, and extension at 72°C for 1 min.

### Immunohistochemical Staining for Thymosin β_10_

Paraffinized human ovarian tumor samples were used for immunohistochemical staining (IHC). The xylene deparaffinized samples were stained with polyclonal thymosin β_10_ antibody (Abcam,). This stain was detected using avidin–biotin–horseradish peroxidase complex (HRP, Abcam). In brief, the deparaffinzed samples were fixed with retrieval solution (citrate buffer; 10 mM sodium citrate buffer, 0.05% Tween 20, pH 6.0) at 95°C for 20 min and subsequently maintained at 4°C for 30 min. The fixed specimen slides were washed three times with 1x PBS, and then incubated with 5% FBS at 25°C for 1 hr. Subsequently, the samples were exposed to polyclonal thymosin β_10_ antibody (1∶50; Abcam) diluted with 3% FBS solution at 4°C overnight, incubated with biotinylated secondary antibody (1∶500) in 3% FBS for 1 hr at room temperature (RT), and further incubated with HRP at 25°C for 1 hr. Finally, the specimen slides were developed using the chromagen 3,3′-diaminobenzidine tetrahydrochloride (DAB; DAKO, Glostrup, Denmark). Cell nuclei were counterstained with Mayer’s hematoxylin (Sigma, St. Louis, MO, USA).

### Immunocytochemical Staining

After exposure to recombinant adenovirus, primary or 2774 ovarian cancer cells were washed twice with PBS and fixed in 4% paraformaldehyde for over 10 min. Then, cells were washed three times in ice-cold PBS, blocked with 3% bovine serum albumin (BSA) in PBS for 1 hr, then incubated with polyclonal anti-thymosin β_10_ (Abcam) or polyclonal anti-human Fas ligand/TNFSF6 antibody (R&D systems, Minneapolis, MN, USA) for 1.5 hr. A further incubation with a secondary Alexa488 anti-rabbit antibody (Invitrogen) was performed for 45 min. The cells were washed three times in ice-cold PBS again and then stained by 4',6-diamidino-2-phenylindole (DAPI, Invitrogen). Finally, the slides were mounted using Crystal/Mount™ reagent (Biomeda Corp, Poster City, CA, USA) and examined for fluorescence using a BX51TR microscope (Olympus, Japan) connected to a digital camera (Olympus, DP50).

### Cell Migration, Invasion, and Wound-healing Assays

After exposure to recombinant adenovirus for 12 hr, 2774 ovarian cancer cells were placed in the upper chambers of a Transwell® plate (Corning Costar, Cambridge, MA, USA) and coated with collagen only or with a collagen matrigel to assess migration and invasion, respectively. Then they were soaked in 1 ml culture media for a further 12 hr. Cells were removed from the upper side of the filter. Those cells that had either migrated to or invaded the underside of the filter were counted with the aid of a microscope. Experiments were performed in^ ^triplicate. In addition to the migration assay using Transwell® plates, recombinant adenovirus-exposed cells were replaced in a 35-mm µ–Dish, low, with a culture insert (Ibidi, Munich, Germany) for 12 hr, and then the direction and speed of cell migration during wound-healing was monitored.

### Cell Viability

Mitochondrial function of cultured 2774 ovarian cancer cells was measured using the 3-(4, 5-dimethylthiazol-2-yl)-2, 5-diphenyltetrazolium bromide (MTT) conversion assay (Mosmann T, 1983). MTT was purchased from Sigma. This assay, which provides a general indication of cell viability, takes advantage of the conversion of the yellow MTT compound to purple formazan crystals by mitochondrial succinate dehydrogenase in viable cells. Briefly, MTT was diluted in 0.1 M phosphate buffered saline (PBS) and added to cells grown in 96-well plates at a final concentration of 0.25 mg/ml. After incubation for 2 hr at 37°C with MTT, the culture medium was removed. The precipitated formazan was solubilized with dimethyl sulfoxide (DMSO) and quantified spectrophotometrically at 595 nm. In addition to the MTT-bio assay for cell viability, the Alamar Blue assay was also performed to quantitatively assess the viability of the 2774 ovarian cancer cells. The oxidized form of Alamar Blue has a dark blue color and little intrinsic fluorescence. When taken into cells, the dye becomes reduced and turns red. This reduced form of Alamar Blue is highly fluorescent. The extent of this conversion, which is a reflection of cell viability, can be quantified by optical density or fluorescence measurements for greater sensitivity. The colorimetric change of Alamar Blue at 570 nm, as compared to the reference wavelength, 600 nm, is proportional to the number of viable cells.

### Annexin V-FITC Flow Cytometric Analysis

The annexin V-FITC apoptosis detection kit (Invitrogen) was used to detect early and late apoptosis. Annexin V has a strong affinity for phosphatidyl serine, which is externalized in the membranes of apoptotic cells. In brief, 2774 ovarian cancer cells were exposed to recombinant adenovirus for 48 hr, washed in PBS, and resuspended in binding buffer (HEPES–NaOH 10 mM pH 7.4, 144 mM NaCl and 25 mM CaCl_2_). Annexin V-FITC (0.2 µg/µl) and propidium iodide (PI, 0.05 µg/µl) were added, and the cells were incubated in the dark for 20 min. Fluorescent activated cell sorting was performed using a FACS Calibur flow cytometer (Becton Dickinson, Franklin Lakes, NJ, USA), and at least 10,000 events were recorded and represented as dot plots.

### Real-time Imaging

Subconfluent co-cultured primary ovarian cancer cells and fibroblasts were grown on a 35-mm confocal dish (SPL, Kyeong-ki, Korea) in the presence of recombinant adenovirus (10 MOI of Ad.TERT.Tβ_10_). The growing cells were photographed every 30 min for 90 hr by means of a live cell confocal microscopy system (Carl Zeiss, Jena, Germany).

### Gel Electrophoresis and Western Blotting

Western blot analysis was performed to determine FAS/CD95 protein expression. After washing 1X with cold PBS, cells were harvested from culture dishes in 200 µl of lysis buffer (Tris 50 mM at pH 7.4, 10 mM EDTA, 1% Tween-20, 10 µM leupeptin, and 1 mM phenylmethyl-sulfonyl fluoride), and the cell lysates were homogenized by a brief sonication. Total cell lysates, containing equal amounts of protein, were subjected to 10% SDS-PAGE at 50 mA and were then transferred onto nitrocellulose membranes for 1.5 hr at 50 V. Non-specific binding was blocked by incubation of the membranes in a solution containing 5% non-fat dried milk in T-TBS buffer (20 mM Tris buffer; 0.5 M NaCl, 0.5% Tween-20). Blots were incubated with polyclonal anti-human Fas Ligand/TNFSF6 antibody (R&D systems, Minneapolis, MN, USA, 1∶1000) followed by incubation with horseradish peroxidase (HRP) antibody (1∶5,000, Abcam). The blots were visualized using the Amersham ECL™ GST Western Blotting Detection reagent and developed using the ECL system (Amersham Pharmacia, Piscataway, NJ, USA).

### High Content Forster Resonance Energy Transfer Screening Analysis

To evaluate the change of mitochondria membrane potential (MMP) activity and caspase 3 activity caused by infection of human ovarian cancer cells with recombinant adenovirus (Ad.TERT.Tβ_10_), we performed high-content Forster resonance energy transfer screening analysis using a Cellomics ArrayScan VTI HCS Reader (Thermo Scientific, Pittsburgh, PA, USA). The MMP and caspase 3 assays were performed according the Thermo Scientific Cellomics HCS Reagent kit instructions (Thermo Scientific). Briefly, various reagents, including ROS inhibitors, a caspase inhibitor, an actin polymerization inhibitor, as well as a recombinant adenovirus, were added to the 2774 human ovarian cancer cells growing on a 96-well plate in DMEM supplemented with 10% fetal bovine serum (FBS). After 48 hr, the cells were washed twice with PBS and stained according to the standard procedure outlined in the Cellomics® HCS Reagent Series Multiparameter Cytotoxicity 2 kit (Thermo Scientific) and the Cellomics® Caspase 3 Activation Kit (Thermo Scientific) to evaluate MMP activity and caspase 3 activity, respectively. Fluorescent cells were photographed at the following wavelengths (excitation/emission): 350/461 nm (Hoechst Dye), 554/576 nm (Mitochondrial Dye), and 562/572 nm (DyLightTM549-Conjugated goat anti-rabbit IgG for Caspase 3). The data were acquired for four fields in each well (n = 6) of the plate using the×20 objective. The data obtained were analyzed using the t-test for independent samples.

### ROS Assay

Intracellular ROS formation was measured by fluorescence using 2′,7′-dichlorofluorescein diacetate (DCF-DA). DCF-DA was purchased from Invitrogen. This nonfluorescent dye is freely permeable to cells, and in cells, DCF-DA is de-esterified to its ionized free acid (DCFH), which then reacts with ROS to form a fluorescent 2′, 7′- dichlorofluorescein dye (DCF). In brief, cells were loaded with 20 µM of DCF-DA and 20% Pluronic F-127 for 30 min, and then kept at room temperature for an additional 30 min to allow for complete dye de-esterification. For DCF measurement, excitation and emission were monitored at 490 nm and 530 nm, respectively, using a fluorescence plate reader (Victor V plate reader, Perkin Elmer, Wellesley, MA, USA).

### Statistical Analysis

The data represent mean ± SE from three independent experiments except where indicated. Data were analyzed using Student’s t-test at a significance level of P<0.01 (**) or P<0.05 (*).

## Supporting Information

Movie S1 **Ad.TERT.Tβ_10_-induced selective apoptotic morphological changes in human primary ovarian cancer cells, but not in normal fibroblasts.** Co-cultured subconfluent primary human ovarian cancer cells and fibroblasts were exposed at 10 MOI of Ad.TERT.Tβ_10_, and morphological changes were observed at every 30 min using a real-time imaging system. White dotted circles are indicating selective morphological features of apoptosis, such as cell plasma membrane blebbing, cytoplasmic vacuolization, and cell shrinkage (after 10 hr), followed by loss of cell-to-cell interactions (after 15 hr). And black dotted circle tracking one of many fibroblasts indicates that they are not only alive but also actively moving for over 90 hrs.
(AVI)
